# Clinical effect of arthroscopic long head of biceps transfer and tenodesis on irreparable rotator cuff tear

**DOI:** 10.1186/s13018-022-03121-5

**Published:** 2022-04-10

**Authors:** Min Ma, Zhangyi Pan, Liangyu Lu

**Affiliations:** Department of Joint and Sports Medicine, East Hospital, Tongji University School of Medicine, 150 Jimo Road, Pudong New Area, Shanghai City, 200120 People’s Republic of China

**Keywords:** Rotator cuff tear, Long head of biceps, Rotator cuff repair

## Abstract

**Objective:**

To explore the clinical effect of arthroscopic long head of biceps transfer and tenodesis for on irreparable rotator cuff tear.

**Methods:**

A total of 18 patients with irreparable rotator cuff tear who were treated in the Dongfang Hospital Affiliated to Tongji University School of Medicine from April 2018 to March 2020 were included in this study. They all underwent arthroscopic long head of biceps transfer and tenodesis. Shoulder joint motions (forward flexion, abduction, and external rotation angle) and magnetic resonance imaging (MRI) were performed. Moreover, visual analogue scale (VAS) and university of California Los Angeles (UCLA) score were conducted during follow-up.

**Results:**

Preoperative symptoms lasted from 3 to 16 months, with an average duration of 10 months. All patients healed in the first stage without obvious complications were included. All patients were followed up for 4 to 14 months after the surgery, with an average duration of 11.1 months. The range of shoulder joint motions, including forward flexion (80.52° ± 31.19° vs. 149.47° ± 28.36°), abduction (65.13° ± 37.59° vs. 152.46° ± 28.64°) and lateral rotation (30.17° ± 15.15° vs. 71.49° ± 11.42°) was significantly improved after operation (*P* < 0.05). The VAS score was notably decreased after operation (8.46 ± 0.80 vs. 1.55 ± 0.70), but the UCLA score was markedly increased (15.27 ± 2.89 vs. 31.17 ± 2.36). MRI imaging showed that 15 patients had good tissue healing, with a healing rate of 83.3% (15/18).

**Conclusion:**

Arthroscopy of the biceps long head tendon transposition can significantly relieve pain in patients with large rotator cuff tears, improve joint mobility, and restore joint function.

## Introduction

Massive rotator cuff tear is one of the severe challenges faced by sports medicine specialists, and its treatment is still controversial [1]. Young patients with acute traumatic rotator cuff tear should be treated by surgery. For the chronic degenerative massive rotator cuff tear that cannot be repaired by standard methods, the treatment options have been concerned by many scholars. Compared with small rotator cuff tear, these "irreparable" massive rotator cuff tears have higher re-tear rate after repair. It has been reported that the treatment failure rate of massive rotator cuff injury is as high as 40% [2].

There is still no consensus on the treatment of irreparable massive rotator cuff tear. The treatment methods include arthroscopy, partial rotator cuff repair, tendon transposition, traditional superior-capsular reconstruction (SCR), and long head of biceps tendon (LHBT) fixation facilitating the upper joint capsule reconstruction (Chinese SCR). The "Chinese SCR" is a method that uses LHBT transposition and fixation on the footprint area to reconstruct the SCR to repair the huge rotator cuff [3]. In the study, we aimed to explore the clinical effect of arthroscopic long head of biceps transfer and tenodesis for on irreparable rotator cuff tear.

## Subjects and methods

### Subjects

This study was in accordance with the Helsinki Declaration and was approved by the ethics committee of the Dongfang Hospital Affiliated to Tongji University School of Medicine (TJDXDFYY-18-0251). Written informed consent was obtained from each participant. A total number of 18 patients who underwent the "Chinese SCR" repair of rotator cuff tears from April 2018 to March 2020 in our hospital were enrolled in this study. Inclusion criteria: (1) Preoperative imaging showed a massive rotator cuff tear before or after the upper rotator cuff [4]; (2) arthroscopy confirmed a massive rotator cuff tear severely retracted to the edge of the glenoid, so that it was impossible to pull back the footprint area, and the subganglion tendon and subscapular tendon were partially or completely torn; (3) the LHBT was continuous and complete, and the quality of the tendon was good, which can be accompanied by partial wear or mild degeneration; (4) conservative treatment was ineffective.

Exclusion criteria: (1) Preoperative imaging showed severe osteoarthritis; (2) LHBT dislocation was found under arthroscopy; (3) rheumatoid or rheumatoid arthritis; (4) pigmented villous nodular synovium inflammation; (5) shoulder or acromioclavicular joint dislocation; (6) severe SLAP injury; (7) previous history of other shoulder surgery or joint infection.

### Surgical methods

After general anesthesia, the patient was placed in the supine lateral position. The traction of the affected limb was 45° abductions, with 20° forward flexion. Traction of 4 to 6 kg was selected, and a 30° arthroscopy was placed in the conventional posterior approach of the shoulder joint. We then established an anterior approach externally and internally at the rotator cuff space and placed the relevant surgical instruments. Routine arthroscopy was used to detect the joint cavity, joint damage, and continuity and quality of the biceps tendon. Next, we implemented a lateral access approach to the shoulder joint and anterolateral approach, placed the arthroscopy into the subacromial space, cleaned the acromion sac, shaped the acromion, and then started the rotator cuff repair operation.

**Fig. 1:**
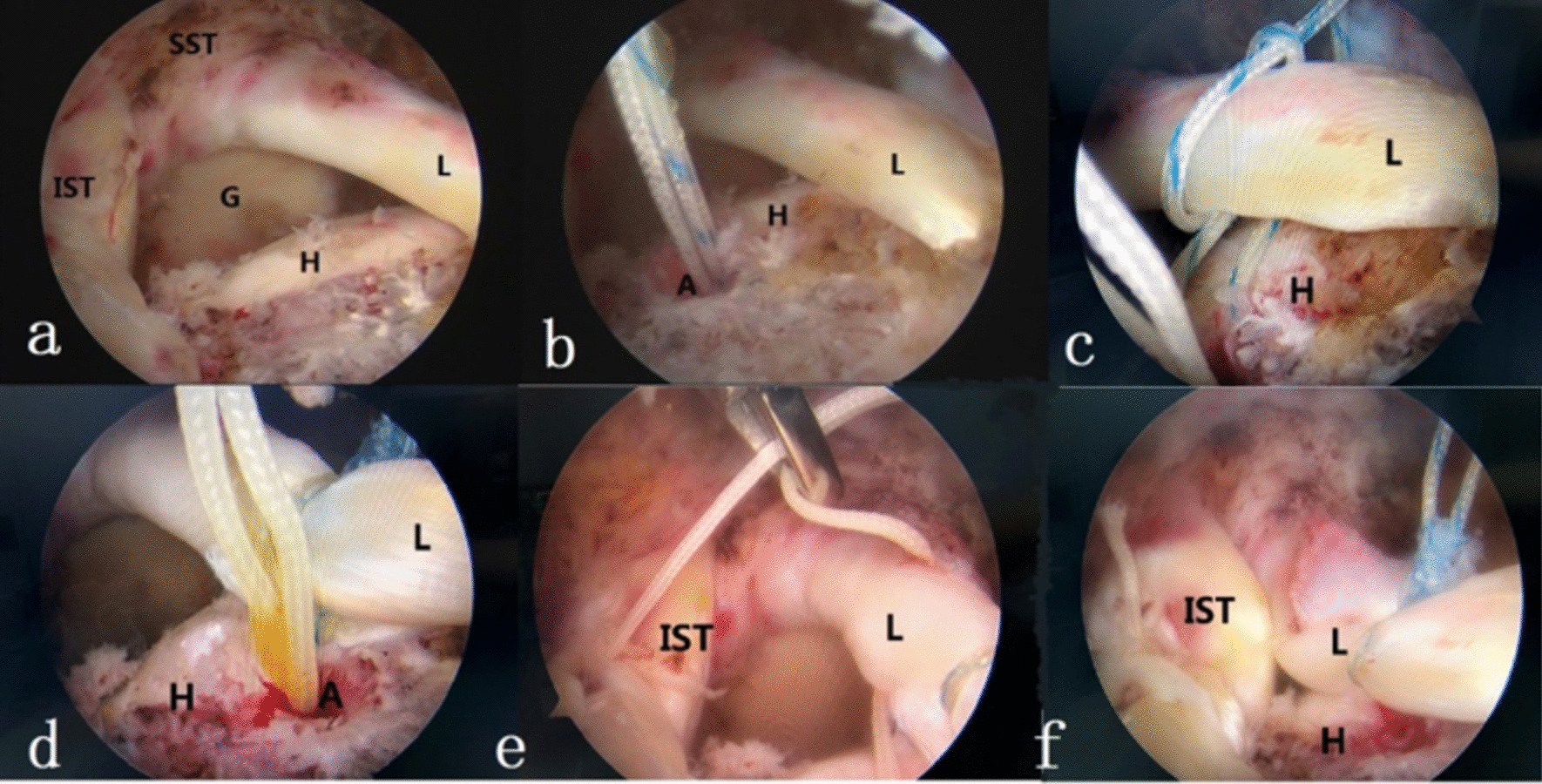
Surgical methods. **a** The supraspinatus tendon completely teared and retracted with the long head tendon of biceps intact; **b** a suture anchor was inserted at the greater tuberosity to prepare for transposition of the long head tendon; **c** pass the blue thread through the long head tendon and knot it with Lasso technique; **d** the long head transformation finished; **e** white suture was passed through the longhead tendon and infraspinatus tendon; **f** white suture knotted, then the closure of rotator cuff defect. *A* anchor with sutures (white and blue); *L* long head of biceps; *H* humrus head; *G* Glenoid; *SST* Supraspinatus tendon; *IST* Infraspinatus tendon

First, we observed the arthroscopic lateral approach to evaluate the rotator cuff as massive tear, involving more than two tendons and obvious retraction of the supraspinatus tendon. It was still difficult to pull back the footprint area after loosening. Although the footprint area could be pulled back, the tension was too large, and there was no obvious tear or degeneration of the LHBT (Fig. 1a). Then, we prepared the rotator cuff footprint area. LHBT was translocated to the near articular surface of the supraspinatus tendon footprint area under appropriate tension. A 4.5-mm PEEK wire anchor (Smithnephew, double wire anchor) was implanted, and Lasso technology fixed displacement LHBT (Fig. 1b–d). (3) We sutured the infraspinatus tendon and LHBT (Fig. 1e, f). After the operation was finished, the shoulder joint brace was kept in a neutral 30° abduction.

### Postoperative rehabilitation

All patients were instructed to perform functional exercises by a rehabilitation specialist who had specialized in sports rehabilitation after surgery. After the surgery, the upper arm was immediately placed at 30° abduction for 6 weeks. On the first day after the operation, the active mobility training of the elbow, wrist, and hand was conducted. Meanwhile, strength training of the upper arm muscles, scapular stability exercises (raising the chest, shrugging the shoulders, and around the shoulders), and shoulder muscle relaxation training (pendulum and circle movement) were performed by goniometer. Shoulder forward flexion and external rotation passive mobility training were done on the first day after the surgery. Ice was placed on the affected area at the end of the exercise to reduce swelling and inflammation (10 °C for 15 min/time). The full joint mobility was restored after six weeks, and active joint mobility training was started at the two months. The muscle strength training around the shoulder joints would be started after three months, and the adversarial training would be performed at the three months.

### Observation indicators

The follow-up was performed by a non-surgical person, and the following criteria were evaluated. Preoperative and postoperative shoulder joint motion (flexion, abduction, and lateral external rotation angle) was examined. Visual analogue scale (VAS) and University of California Los Angeles (UCLA) score were assessed. Availability of tympanic deformity, chronic pain, joint adhesion, infection, and anchor complications such as nail displacement or prolapse were assessed. Magnetic resonance imaging (MRI) examination was performed before and after surgery (3 months after surgery) to observe the position of the humeral head and reconstruct the tissue integrity.

### Statistical analysis

Statistical analysis was made by software SPSS17.0 (International Business Machines, corp., Armonk, NY, USA). Significant differences before and after the operation were assessed by Wilcoxon rank sum test. All data were expressed as means ± standard deviation (SD). *P* < 0.05 was considered statistically significant.

## Results

### General information

There were 7 males and 11 females, with the average age of (63 ± 24) years. Their preoperative symptoms lasted for 3 to 16 months, with an average of 10 months.

### Complications

All patients were followed up for 4 to 14 months, with an average of 11.1 months. All patients healed in the first stage without joint adhesion, infection, and postoperative anchorage were included. There were no obvious complications and adverse reactions such as displacement or prolapse.

### Joint motion

The data of the shoulder joint mobility before and after operation are presented in Table 1. The postoperative forward flexion, abduction, and lateral rotation were significantly improved after operation (*P* = 0.00).

**Table 1:** Comparison of shoulder range of motion before and after operation

Items	n	Forward flexion	Abduction	Lateral rotation
Pre-operation	18	80.52° ± 31.19°	65.13° ± 37.59°	30.17° ± 15.15°
Final follow-up	18	149.47° ± 28.36°	152.46° ± 28.64°	71.49° ± 11.42°
*P*		0.00	0.00	0.00

### Pain and function scores

The VAS and UCLA scores before and after operation are shown in Table 2. All patients' postoperative scores were significantly improved (*P* = 0.00). The UCLA score was superior in 5 patients at the last follow-up. Ten patients had good scores, whereas 3 patients obtained poor scores. The rate of the excellent and good scores was 83.3% (15/18).

**Table 2:** Comparison of shoulder pain and function scores before and after operation

Items	n	VAS	UCLA
Pre-operation	18	8.46 ± 0.80	15.27 ± 2.89
Final follow-up	18	1.55 ± 0.70	31.17 ± 2.36
*P*		0.00	0.00

### MRI review

**Fig. 2:**
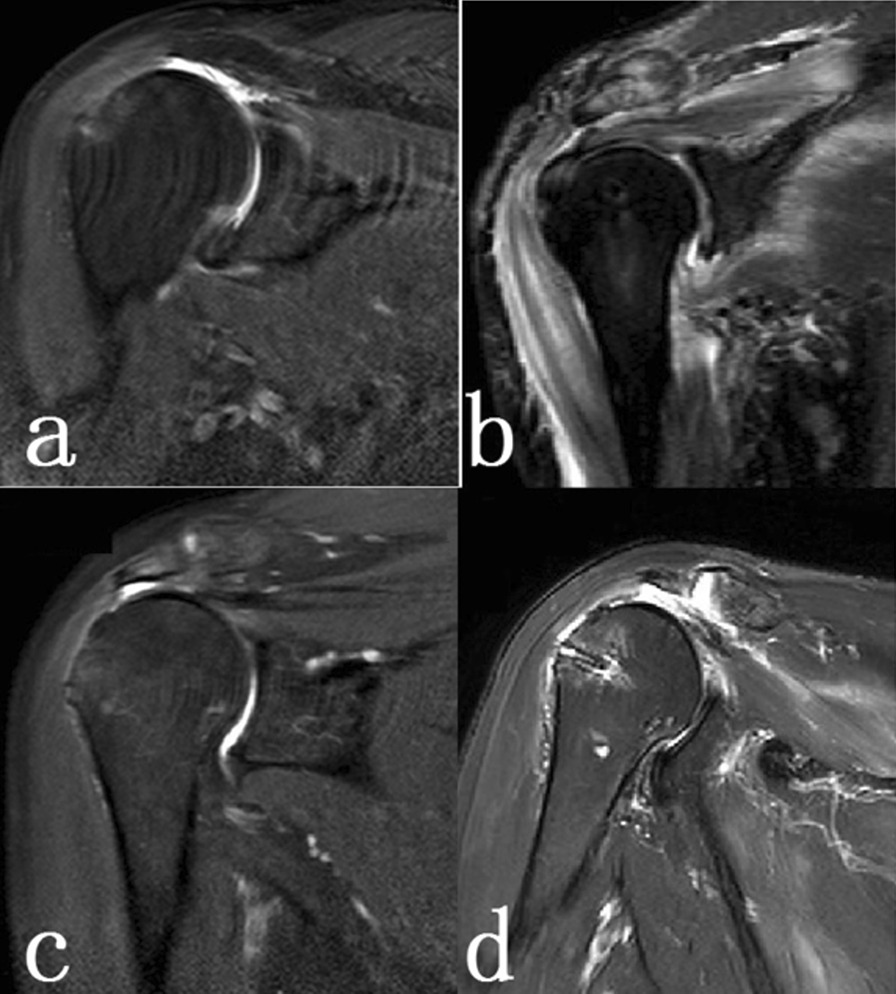
MRI imaging of shoulder reconstruction. **a** Preoperative complete structural continuity; **b** postoperative complete structural continuity; **c** preoperative structural failure; **d** postoperative structural failure

Postoperative MRI was reexamined 3 months after operation. At the final follow-up, MRI imaging showed that 14 patients had completely reconstructed tissue structure, with a healing rate of 77.8% (14/18). However, 4 patients had obvious structural failure (Fig. 2).

## Discussion

With the improvement of the rotator cuff repair and the development of shoulder arthroscopy technology, satisfactory outcomes have been achieved after arthroscopic repair of rotator cuff tears with small and medium degrees. However, no curative effects of repair of massive rotator cuff tear have been achieved. The re-tear rate is high, with highest reported in the literature of approximately 90% [5–11]. For example, Chung et al. performed arthroscopy on 108 patients with massive rotator cuff tear, and they found that 39.8% of patients had recrudescence [12]. Currently, no unified understanding exists in the treatment of massive irreparable rotator cuff tears. The commonly applied methods include arthroscopy and subacromial decompression, LHBT cut or fixation, partial rotator cuff repair, patch enhancement, tendon transposition, reverse shoulder replacement, etc. Joint cleanup and LHBT amputation or fixation can significantly improve the patient's pain and other symptoms [13, 14], with partial and complete repair of the rotator cuff. However, compared with postoperative pain, poor functional improvement has been previously noted. For example, Franceschi et al. found that the average UCLA and RC-QOL scores of the joint cleaning group were lower than those of the partially repaired group [15, 16]. The researchers also established that the improvement of the Constant–Murley score of the joint cleanup and LHBT resection was significantly lower than that of the rotator cuff repair. Although some repairs can achieve better functional improvement in the short term, the long-term efficacy is still not good [17, 18]. Mihata et al. first proposed the use of autologous broad fascia as a repair material for SCR treatment of huge irreparable rotator cuff tears [19–21]. The postoperative follow-up showed that it significantly improved the patient's functional score, and satisfactory results were achieved. However, SCR is traumatic and costly, and it usually requires at least six anchors to fix the broad fascia, and the economic burden is heavy. It is noteworthy that the application of rotator cuff patch to massive rotator cuff tear has also achieved encouraging results. However, it also faces problems such as high cost, difficulty in achieving integration between the patch and rotator cuff, degradation of the patch, and other complications, which limit its application [16, 22, 23].

Here, we reported a new treatment method, in which we use LHBT during surgery. In this new approach, we retained the LHBT, laterally transferred to the supraspinatus tendon footprint area for fixation to simulate the SCR and bridge the partially repaired rotator cuff. The underlying principle of this method is the application of LHBT to provide a reduced brace to increase the mechanical strength of the front rotator cuff and the upper blocking effect to assist the repair of the huge rotator cuff. Reduction of the tension of the suturing rotator cuff tissue is also achieved. This method not only prevents the auto-graft material, but also allows for the use of a reduced number of anchors. The described method requires only 1–2 anchors, which greatly reduces the cost of surgery. The cases included in this study were patients with a completely torn superior tendon, and the footprint area was difficult to pull back. This situation was particularly suitable for LHBT transposition for SCR. The VAS score and UCLA scores and joint mobility were significantly improved than pre-operation, and no complications such as joint adhesions occurred. Imaging revealed that 77.8% (14/18) of the reconstructed tissue structure was intact.

El shaar [24] randomly divided 10 cadaveric shoulder joint specimens into two groups, 5 in each group. Supraspinatus tendon was cut off in the foot print area of the greater tuberosity, and then SCR was performed with autologous LHBT and autologous fascia. Their results showed that the LHBT autologous graft SCR could obtain the same or even stronger biomechanical stability as the autologous fascia SCR in preventing the humeral head from moving up. Han et al. [25] also found that shifting LHBT to the humeral head for SCR can reduce the humeral head uplift caused by huge rotator cuff tear. Importantly, LHBT can achieve the same biomechanical effect whether it is sutured with the subganglar tendon or not.

However, few clinical studies on LHBT displacement have been reported. Cho et al. [26] divided 68 patients with massive rotator cuff tears into two groups. 37 patients underwent LHBT enhanced repair (group A), and 31 patients underwent conventional rotator cuff repair (group B). They found that the UCLA function scores of group A and group B increased from 14.1 and 13.9 points before surgery to 32.6 and 30.3 points after surgery, respectively. The forward flexion, external rotation, and internal rotation muscle strength in group A were more significantly improved than those in group B. MRI scans revealed that the healing rate of rotator cuff in group A was significantly higher than that in group B. It showed that compared with conventional repair methods, LHBT enhanced repair can significantly reduce the failure rate and increase muscle strength. Unlike the method of Cho et al., we did not cut off the LHBT glenoid joint but only fixed the LHBT displacement, thus retaining the blood supply at the proximal end of the LHBT. No significant complications and adverse reactions were observed during follow-up. However, because of the small sample size included in this study and the short follow-up time, study with long-term follow-up and large sample size is still needed.

## Conclusion

Arthroscopy of the biceps long head tendon transposition can significantly relieve pain in patients with large rotator cuff tears, improve joint mobility, and restore joint function.

## Data Availability

Data sharing is not applicable to this article as no datasets were generated or analyzed during the current study.
